# The Woman’s Dental Association of the United States

**Published:** 1894-01

**Authors:** 


### The Woman's Dental Association of the United States.

The meeting of the Woman's Dental Association of the United States was held in the Memorial Art Palace, Friday, August 18, 1893, commencing at 10:30 A. M. Between thirty-five and forty women dentists were present.

In the absence of the President, Dr. Mary H. Stillwell, the meeting was called to order by Dr. Emma L. Benham, and on motion, Dr. Elizabeth Davis, Philadelphia, Chairman of the Executive Committee, was called upon to preside.

The Secretary, Dr. Eliza Yerkes, read a letter from the President, expressing her regret at her unavoidable absence. The President spoke of the apathy of women practitioners toward any concerted efforts for their own advancement, but the work of forming an association of women dentists had been accomplished, and the movement would doubtless prove of the greatest advantage to all women practitioners.

The Corresponding Secretary, Dr. Anna T. Focht, Philadelphia, in her annual report, laid great stress on the fact that great indifference had been exhibited in the matter of replies to letters and circulars sent out from her office.

A brief review of the history of the National Association was given by the Recording Secretary. The first meeting looking toward such an organization was held March 19, 1892, and a charter was obtained in July, 1892. At the present date there is a membership of thirty-three registered women dentists, with six applications under consideration.

The Treasurer's report for the year showed a small balance on hand.

The roll of States represented in the association was then called for, with reports from the several Vice-Presidents.

The Vice-President from the State of Illinois reported that there are in the State five colleges recognized by the National Association of Dental Examiners as *reputable.* Unfortunately, two of these refuse to admit women as students. There are eight registered women practitioners in Chicago, one in Springfield, and probably others in the State. Woman's work in dentistry is recognized as creditable and satisfactory.

The officials of the World's Columbian Dental Congress have shown the most favorable disposition toward women dentists, but the women themselves have been slow to respond. Women physicians and women dentists work harmoniously together, especially since the more general recognition of the reflex of the fifth nerve. Dentistry is recognized as a specialty of medicine, and women dentists must make every effort to rise proportionately. I hope, then, that our women will come forward and take hold of society work, the surest method of self-elevation.

The Vice-President from Massachusetts said she only knew of five women practitioners in her State. The Boston Dental College admits women students on an equality with men. Harvard does not yet admit us, but one of their professors said to me, "You are knocking at the door so loudly that we have got to do it."

The representative from Maryland said that she occupied the forlorn position of being the only woman practitioner in that State. Women were not admitted to the colleges nor to the societies, except on one occasion she had been offered a position on a committee requiring the payment of a fee.

The Vice-President from Washington, D. C., sent the sad message that she was writing from the bedside of a dying father, and could furnish no report.

The representative from Rhode Island said that she also claimed fellowship with Massachusetts, as she was a graduate of the Boston Dental College. She was a member of the State Society, and had never met with any professional opposition in either State.

The representative from Wisconsin stated that six or seven ladies were practicing dentistry in that State, and they seemed to be gladly welcomed by the people.

The Corresponding Secretary now read a most interesting letter from Prof. Jas. Truman, relating the struggles marking the admission of women into the dental colleges. At the conclusion of the reading, on motion of Dr. Hirschfeld, the first woman graduate in dentistry, a rising vote of thanks was tendered Dr. Truman, in recognition of his services in the cause of woman in dentistry.

Dr. Truman responded with great feeling to this mark of appreciation.

The Chairman of the Association hoped that some action would be taken to give the letter of Dr. Truman greater publicity than would be secured by its publication in the annual transactions of the Association, which would reach only the members of the society, and a committee was therefore appointed to see that proper publicity be secured.

Dr. Benham thought that as a history of women in dentistry it should have a place in the transactions of the Congress, beside that of Dr. Shepard giving the history of what man has done in dentistry. The photographs of Dr. Truman and Dr. Taft were also desired for a place in the transactions of the Society. It was stated that the Baltimore College of Dental Surgery had graduated eight or ten women while Prof. Gorgas was Dean of that institution.

The question was asked as to the number of women graduates in dentistry. The reply was made that the present location of about one hundred and fifty was known, though there were more than that number, as many had made no response to letters, which, however, had not been returned to the writer. The colleges know how many they have graduated, but they cannot keep track of their locations.

Certificates were read showing that Mrs. E. R. Jones had, prior to 1859, assisted her husband in his dental practice, and had continued it after his death.

The recognition of the society was given to the labors of Dr. Hattie E. Lawrence in forwarding the interests of the present meeting.

The representatives from Colorado, Missouri, Montana, and Nebraska presented their respective individual reports, and Mrs. J. M. Walker spoke for the women of the far South.

A message of greeting to the absent President was voted, and the Association then adjourned.

